# Rational prescribing of antibiotics in children under 5 years with upper respiratory tract infections in Kintampo Municipal Hospital in Brong Ahafo Region of Ghana

**DOI:** 10.1186/s13104-018-3542-z

**Published:** 2018-07-04

**Authors:** Abdul-Nasiru Sumaila, Philip Teg-Nefaah Tabong

**Affiliations:** 1 Pharmacy Department, Jema District Hospital, Ghana Health Services, Accra, Brong Ahafo Region Ghana; 2 Department of Social and Behavioural Sciences, School of Public Health, University of Ghana, Legon, Accra, Ghana

**Keywords:** Rational, Medicine, Antibiotics, Children, Upper respiratory tract infection, Ghana

## Abstract

**Objective:**

The aim of the study was to assess the rational use of antibiotics in children with URTIs in the Kintampo Municipal Hospital in Ghana.

**Results:**

A total of 839 medicines were prescribed, 237 were antibiotics. The mean number of medicines prescribed per patient encounter was 3.1. The percentage of patient encounters with antibiotics was 28.2 and 0.4% for injections. The percentage of medicines prescribed by generic was 93.8% and from the essential medicines list was 94.9%. Ninety-two of patients received amoxicillin. Polypharmacy was common as prescriptions with five to six medicines per patient encounter was found. Some prescribers are not following the WHO/INRUD requirement of prescribing medicines in their generic and from the essential medicine list of the country.

## Introduction

In the last decades, medicines have had an unprecedented positive effect on health, leading to decline in mortality, disease burden and overall quality of life [1]. The rational use of medicines is regarded as a measure of good clinical practice. The inappropriate use of medicines especially antibiotics have negative consequences on the quality of care and can lead to antibiotic- resistance strains of micro-organisms [2–5]. The conference of experts on the rational use of medicines (RUM), convened by the World Health Organization (WHO) in Nairobi in 1985 defined the RUM as giving patients medicines that are appropriate to their clinical needs, in doses that meet their own individual requirements for an adequate period of time, and at the lowest cost to them and their community. In view of this, the use of medicines that do not meet the needs of patients in terms of disease condition, dose, frequency and duration of therapy is described as inappropriate use of medicines [2]. This notwithstanding, studies have showed a high prevalence of antibiotic use among children under 5 with an estimate rate of 2.2 prescriptions per person per year [6, 7]. Though there are no age-specific disaggregated data, earlier studies have found that antibiotic use in Ghana to be between 11.9 and 60.7% [8–10].

Upper respiratory tract infections (URTIs) are infectious diseases of the upper respiratory tract and include condition such as common cold, influenza, pharyngitis, otitis media, tonsillitis and sinusitis [11]. However, common cold is reported as the most prevalent accounting for about 80% of URTIs [12]. URTIs are mostly managed symptomatically with basic analgesics to relief fever, increased fluid intake and with nasal decongestants [13] because they are mostly viral in origin and many (about 90%) resolve without any intervention [12].

URTIs are very common among paediatric population and therefore constitute a major target for inappropriate use of antibiotics. As a measure to help reduce the inappropriate use of antibiotics, the WHO has advocated for the use of essential medicine list and other policy guidelines for the use of medicines including antibiotics [14–16]. The Ghana National Drug Programme has developed and distributed the essential medicines list (EML) and Standard Treatment Guidelines (STGs) to all public health institutions over the years [17]. The national drug treatment guideline requires that all prescription should be generic and should be listed in the essential medicine list of the country [17]. The STGs provides for age and disease-specific doses and duration of treatment. The aim of this study was to assess rational use of medicine among paediatric patients in the Kintampo Municipal hospital.

## Main text

### Materials and methods

#### Study design

We reviewed medical records of children under 5 years who attended the out-patient department of Kintampo Municipal Hospital with URTIs between the 1st of January, 2009 and 31st December, 2014 using a WHO checklist on rational use of medicine.

#### Study area

The Kintampo Municipality is one of the 28 districts in the Brong Ahafo region of Ghana. The municipality has a population of 111,263 [18]. The Kintampo Municipality has one District hospital of 125 bed capacity, two Health centres, two Rural clinics, one Community-based Health Planning Services (CHPS) compound and one Maternity home.

#### Sample size determination

We used Yamane’s formula (1967) for population proportion to compute the sample size for this study;

1 $${\text{n}} = \frac{N}{{1 + N(e^{2} )}}$$

where n = minimum sample size, N = population size of 648, e = level of precision which was set at 5%. Substituting the above into Eq. 1 resulted in a sample size of 248. However, it was increased by 10% to 270.

#### Data collection

A systematic random sampling procedure was used. The sampling interval of two was obtained by dividing the study population (N) by the sample size (n) [19]. We used this sampling interval to sample patients and retrieved their medical records for the review.

#### Study variables

We extracted the following variables from patients primary medical records: diagnoses (primary and secondary) according to the International Classification of Diseases (ICD-9), age, sex, and prescription of antibiotic at that visit (whether prescribed or not, and if so, the type of antibiotic selected), dose, frequency and duration.

#### Data analysis

The data extracted were entered into STATA 13 and analysed. The results were than compared with WHO benchmark indicators. Appropriateness of treatment was determined by the extent of adherence to the Ghana STG and EML [20] using the criteria recommended by Kunin et al. [21] and Deshmuch et al. [22].

### Results

#### Type of URTIs and antibiotics used

The minimum age was 0.5 months (15 days), the maximum age was 57 months with an average age of 18.3 ± 13 months. Children aged 1–5 years (12–59 months) accounted for majority of patients (62.6%). Only 3 patients were less than 1 month (1.1%). Out of the 270 patients, 140 (52.0%) were males. Among the 270 patients, the most common URTI was common cold (52.6%). However, 17 patients had conditions which did not fit into the classification criteria for any URTIs and were therefore described as non-specific.

A total of 839 medicines were prescribed for the 270 patients with no patient reporting multiple attendance for URTI. Two hundred and seven (237) antibiotics were prescribed per patient encounter; penicillin (58%), cephalosporins (19%) and macrolides (10%). Amoxicillin (38.8%), cefuroxime (18.6%) and amoxicillin + clavulanic acid (17.3%) were the commonly prescribed antibiotics (Table 1).

**Table 1:** Antibiotics prescribed for the management of URTIs

Antibiotic (n = 237)	Frequency (F)	Percentage (F × 100/n)
Penicillins		
Amoxicillin suspension	92	38.8
Amoxicillin + clavulanic acid suspension	41	17.3
Flucloxacillin suspension	4	1.7
Cephalosporins		
Cefuroxime suspension	44	18.6
Ceftriaxone injection	1	0.4
Macrolides		
Azithromycin suspension	17	7.2
Erythromycin suspension	7	3.0
Sulphonamides		
Cotrimoxazole suspension	21	8.9
Others	10	4.2
No antibiotic	33	12.2

Sixty-two (43.7%) of patients with common cold received amoxicillin, 5 (3.5%) received amoxicillin with clavulanic acid and 6 (4.2%) received azithromycin and cefuroxime whilst 32 (22.6%) did not receive any antibiotic. Among patients with otitis media, majority, 23 (33.3%) were given amoxicillin + clavulanic acid whilst 21 (30.4%) received amoxicillin. Patients with pharyngitis received amoxicillin with clavulanic acid (47.8%).

#### Assessment of rational prescribing

The mean number of medicines per patient encounter was 3.1 which was higher than the WHO standard of ≤ 2 medicines per patient encounter. The prevalence of antibiotic use for URTIs was 28.6%. Prescription by generic and from the essential medicines list were 93.8 and 94.9%, respectively (Table 2).

**Table 2:** Comparison between the uses of medicine for URTIs in the study are against the WHO standard

Prescribing indicator	Value	WHO standard
Total number of patients prescriptions analysed	270	≥ 100
Total number of medicines prescribed	839	–
Average number of medicines prescribed per encounter	3.1	≤ 2
% Medicines prescribed by generic	93.8	100
% Patient encounters with antibiotics prescribed	28.2	≤ 30
% of patient encounters with injections prescribed	0.4	≤ 10
% of medicines prescribed from essential medicines list or formulary	94.9	100

About 93.0% of prescriptions had appropriate doses, 95.6% were appropriate in frequency and 96.7% had appropriate duration of treatment (Table 3).

**Table 3:** Distribution of prescriptions according to appropriateness

Parameter	No. appropriate	Prescriptions (N = 270)		% inappropriate
		% appropriate	No. inappropriate	
Dose	251	93.0	19	7.0
Frequency	258	95.6	12	4.4
Duration	261	96.7	9	3.3

### Discussion

This study revealed that common cold was the most diagnosed URTI, followed by otitis media. Common cold accounted for more than half of the diagnoses of the total prescriptions. This findings as agrees with literature that showed that children tend to develop 3–8 bouts of common cold in a year and this even tends to increase for children who attend daycare or preschool [23, 24]. Out of the 142 URTIs diagnosed with common cold, 110 (77.4%) were given antibiotics. In Northern Tanzania, it was found that about 68.9% of children under 5 received antibiotic [25] despite the fact this condition in mainly viral and therefore the use of antibiotic is inappropriate. Generally, it is recommended that children with common cold should managed with nasal decongestant [26], antihistamine [27] and zinc [28] as these help to relief symptom leading to spontaneous recovery. The use of intranasal ipratropium bromide has also be found to be very useful in relieving symptoms of common cold [29]. The findings of study underscore the need to sensitize prescribers on rational use of medicines especially for common cold.

The study revealed three main classes of antibiotics which were commonly used to manage URTIs in children in the hospital. These classes were the penicillins, cephalosporins and the macrolides and similar findings have been reported in a study in South Western Nigeria [30]. The most prescribed class was the penicillins specifically amoxicillin and amoxicillin + clavulanic acid. Amoxicillin is the first line antibiotic in the management of URTIs according to the Standard Treatment Guideline of Ghana [31].

Furthermore, the study revealed a 3.1 average number of medicines prescribed per patient encounter, implying that, patients were likely to receive three medicines per visit. The WHO indicator tools for rational prescribing however requires that only one or two medicines are prescribed per patient encounter [32]. This finding suggests the presence of poly pharmacy. A study carried out at the Ghana Police Hospital revealed a similar finding of 3.7 [33]. Similar findings have been reported in studies in Uganda (2.6) [34] and Jordan (2.4) [35].

The findings of this study further showed that the percentage of medicines prescribed by generic wad 93.8% which is less than the requirement that all medicines should be prescribed in generic. This further call for refresher training for prescribers to conform to the standards in prescribing medicines for patients as rational use of medicines has become an indicator for measuring quality of health care [36–38].

The percentage of patient encounters with antibiotics prescribed was 28.6%. This is within the range of ≤ 30% optimal, WHO standards and therefore tends to suggest a prudent use of antibiotics in Kintampo Municipal Hospital. Such a judicious use of antibiotics will go a long way to minimize antibiotic resistance and serious adverse effects [39, 40]. A percentage of 0.4% obtained for patient encounters with injections is also within WHO/INRUD Standard of ≤ 10%. This is however higher than the findings of a study conducted in Ethiopia that reported a lower percentage of 0.04% [41]. Globally, the use injections should be kept to the minimum because of the increasing risk of transmission of HIV, hepatitis and other blood related diseases through injection [42–44]. The low rate of injection use is also likely to reduce the risk of anaphylactic shock, tissue necrosis and sepsis in patients [45–47].

The findings showed that 94.9% for medicines prescribed from the essential medicines list falls short of the optimal WHO/INRUD standard of 100%. This is however higher than a study conducted in Ghana in 2014 which found a 53.6% adherence to WHO/INRUD requirement [33]. This finding also indicate the need for training and development of quality assurance programmes. In Europe, quality assurance programmes and public campaigns have been developed and launched to improve the use of antibiotics in primary care [48–50]. This strategy could be harnessed for Ghana to improve rational use of antibiotics and adherence to standards in the use of medicines.

### Conclusions

This study conclude that the overuse of antibiotics for URTIs was common in Ghana. The penicillins and cephalosporins are the most preferred antibiotics prescribed for the management of upper respiratory tract infections in children under 5 years. Some prescribers are not following the WHO/INRUD requirement of prescribing medicines in their generic and from the essential medicine list of the country.

## Limitation

The study was carried out in only one health facility within the municipality and region, hence the conclusions should be interpreted in the context of the limited scope of the research.

## Abbreviations

INRUD: International Network for Rational Use of Drugs
RUM: rational use of medicine
STG: Standard Treatment Guideline
URTI: upper respiratory tract infection
WHO: World Health Organisation
